# Genetic and Molecular Mechanisms Underlying Symbiotic Specificity in Legume-Rhizobium Interactions

**DOI:** 10.3389/fpls.2018.00313

**Published:** 2018-03-09

**Authors:** Qi Wang, Jinge Liu, Hongyan Zhu

**Affiliations:** Department of Plant and Soil Sciences, University of Kentucky, Lexington, KY, United States

**Keywords:** legume, nodulation, nitrogen fixation, rhizobial symbiosis, host specificity

## Abstract

Legumes are able to form a symbiotic relationship with nitrogen-fixing soil bacteria called rhizobia. The result of this symbiosis is to form nodules on the plant root, within which the bacteria can convert atmospheric nitrogen into ammonia that can be used by the plant. Establishment of a successful symbiosis requires the two symbiotic partners to be compatible with each other throughout the process of symbiotic development. However, incompatibility frequently occurs, such that a bacterial strain is unable to nodulate a particular host plant or forms nodules that are incapable of fixing nitrogen. Genetic and molecular mechanisms that regulate symbiotic specificity are diverse, involving a wide range of host and bacterial genes/signals with various modes of action. In this review, we will provide an update on our current knowledge of how the recognition specificity has evolved in the context of symbiosis signaling and plant immunity.

## Introduction

The legume-rhizobial symbiosis starts with a signal exchange between the host plant and its microsymbiont (Oldroyd, 2013). Recognition of compatible bacteria by the host induces cortical cell divisions to form root nodule primordia, and simultaneously initiates an infection process to deliver the bacteria into the nodule cells. Infection of most legumes involves the development of plant-made infection threads that initiate in the root hair. The infection threads harboring dividing bacteria grow through the epidermal cell layer into the nodule cells, where the bacteria are released and internalized in an endocytosis-like process. In nodule cells, individual bacteria are enclosed by a membrane of plant origin, forming an organelle-like structure called the symbiosome, within which the bacteria further differentiate into nitrogen-fixing bacteroids (Jones et al., 2007; Oldroyd et al., 2011).

Symbiotic nodule development involves synchronous differentiation of both nodule and bacterial cells. Legume nodules can be grouped into two major types: indeterminate (e.g., pea, clovers, and *Medicago*) and determinate (e.g., soybeans, common bean, and *Lotus*) (Nap and Bisseling, 1990; Hirsch, 1992). Indeterminate nodules originate from cell divisions in the inner cortex and possess a persistent apical meristem. Consequently, indeterminate nodules are cylindrical in shape, with a developmental gradient from the apex to the base of the nodule, which can be divided into different nodule zones (Nap and Bisseling, 1990). In contrast, determinate nodules result from cell divisions in the middle/outer cortex of the root, lack a persistent meristem, and are spherical in shape. Cell divisions of a determinate nodule cease at early developmental stages and the mature nodule develops through cell enlargement; as such, the infected cells develop more or less synchronously to the nitrogen-fixing stage. In both nodule types, the symbiotic nodule cells undergo genome endoreduplication, leading to polyploidization and cell enlargement. Parallel to the nodule cell development is the differentiation of the nitrogen-fixing bacteroids. Depending on the host, but independent of the nodule type, such bacterial differentiation can be terminal or reversible. Terminal differentiation is featured by genome endoreduplication, cell elongation, increased membrane permeability, and loss of reproductive ability, while in reversible differentiation the bacteroids retain cell size and DNA content similar to free-living bacteria (Kereszt et al., 2011; Oldroyd et al., 2011; Haag et al., 2013). Compared to free-living bacteria, the bacteroids display dramatic changes in transcriptome, cell surface structure and metabolic activities so that they become better adapted to the intracellular environment and dedicated to nitrogen fixation (Mergaert et al., 2006; Prell and Poole, 2006; Haag et al., 2013).

Both legumes and rhizobial bacteria are phylogenetically diverse. No single rhizobial strains can form symbiosis with all legumes, and vice versa. Specificity occurs at both species and genotypic levels (Broughton et al., 2000; Perret et al., 2000; Wang et al., 2012). This can take place at early stages of the interaction so that the same bacterial strains can infect and nodulate one host plant but not another (Yang et al., 2010; Wang et al., 2012; Tang et al., 2016; Fan et al., 2017). Incompatibility also frequently happens at later stages of nodule development such that nitrogen-fixing efficiency differs significantly between different plant-bacteria combinations (Wang et al., 2012, 2017, 2018; Yang et al., 2017). Symbiotic specificity results from the changing of signals from both host and bacterial sides; as such, various recognition mechanisms have evolved during the process of co-adaptation. Knowledge of the genetic and molecular basis of symbiotic specificity is important for developing tools for genetic manipulation of the host or bacteria in order to enhance nitrogen fixation efficiency. In this review, we will discuss our current understanding of the evolution of specificity in the root nodule symbiosis.

## Specificity Mediated by Flavonoids and the Flavonoid-NodD Recognition

Under nitrogen-limiting conditions, legume roots secrete a cocktail of flavonoid compounds into the rhizosphere, and they serve to activate the expression of a group of bacterial nodulation (*nod*) genes, leading to the synthesis of the Nod factor, a lipochitooligosaccharidic signal that is essential for initiating symbiotic development in most legumes (Oldroyd et al., 2011). Induction of *nod* gene expression is mediated by the flavonoid-activated NodD proteins, which are LysR-type transcription regulators (Long, 1996). NodDs activate *nod* gene expression through binding to the conserved DNA motifs (*nod* boxes) upstream of the *nod* operons (Rostas et al., 1986; Fisher et al., 1988).

NodD proteins from different rhizobia are adapted to recognizing different flavonoids secreted by different legumes, and this recognition specificity defines an early checkpoint of the symbiosis (Peck et al., 2006). Despite the absence of direct evidence for physical interaction between the two molecules, flavonoids have been shown to be able to stimulate the binding of NodD to *nod* gene promoters in *Sinorhizobium meliloti* (Peck et al., 2006). It is well documented that inter-strain exchange of *nodD* genes can alter the response of the recipient strain to a different set of flavonoid inducers and hence the host range (Horváth et al., 1987; Perret et al., 2000). For example, the transfer of *nodD1* from the broad host range symbiont *Rhizobium* sp. NGR234 to the restricted host range strain *Rhizobium leguminosarum* biovar *trifolii* ANU843 enabled the recipient strain to nodulate the non-legume *Parasponia*, because the wide-host-range NodD1 protein is capable of recognizing a broader spectrum of flavonoid inducers (Bender et al., 1988).

The evidence for the importance of flavonoids in determining host range primarily comes from bacterial genetics, and the plant genes involved are less studied. Since legume roots secrete a complex mixture of flavonoid compounds, it is difficult to pinpoint which flavonoids play a more critical role, and when and where they are produced. Recent studies in soybeans and *Medicago truncatula* have highlighted key flavonoids required for rhizobial infection (reviewed in Liu and Murray, 2016). These so called “infection flavonoids” are strong inducers of *nod* genes, secreted by roots, highly accumulated at the infection sites, and show increased biosynthesis in response to infection by compatible rhizobia. Although luteolin was the first flavonoid identified that can induce *nod* gene expression across a wide range of rhizobial strains, it is not legume-specific, mainly produced in germinating seeds, and has not been detected in root exudates or nodules. In contrast, methoxychalcone has been shown to be one of the strong host infection signals from *Medicago* and closely related legumes that form indeterminate nodules, while genistein and daidzein are crucial signals from soybeans that form determinate nodules. Part of the flavonoid compounds may also function as phytoalexins, acting to reinforce symbiosis specificity (Liu and Murray, 2016). For example, *Bradyrhizobium japonicum* and *Mesorhizobium loti*, but not the *Medicago* symbiont *S. meliloti*, are susceptible to the isoflavonoid medicarpin produced by *Medicago* spp. (Pankhurst and Biggs, 1980; Breakspear et al., 2014), and the soybean symbionts *B. japonicum* and *S. fredii* are resistant to glyceollin when exposed to genistein and daidzein (Parniske et al., 1991).

## Specificity Mediated by Nod-Factor Perception

Nod factors produced by rhizobia are an essential signaling component for symbiosis development in most legumes. Nod factors are lipochito-oligosaccharides, consisting of four or five 1,4-linked *N*-acetyl-glucosamine residues that carry a fatty acyl chain of varying length attached to the C-2 position of the non-reducing end and various species-specific chemical decorations at both the reducing and non-reducing ends (Dénarié et al., 1996). The common *nodABC* genes contribute to the synthesis of the chitin backbone, while other strain-specific *nod* genes act to modify the backbone by changing the size and saturation of the acyl chain, or adding to the terminal sugar units with acetyl, methyl, carbamoyl, sulfuryl or glycosyl groups. Structural variations in Nod factors are a key determinant of host range, because these Nod factors have to be recognized by the host in order to initiate infection and nodulation (Perret et al., 2000; D’Haeze and Holsters, 2002).

Nod factors are perceived by Nod-factor receptors (e.g., NFR1 and NFR5 in *Lotus japonicus*), which are LysM-domain-containing receptor kinases (Limpens et al., 2003; Madsen et al., 2003; Radutoiu et al., 2003). Direct binding of Nod factors to the extracellular LysM domains of the receptor complex leads to activation of the downstream nodulation signaling pathways (Broghammer et al., 2012). Specificity in Nod-factor binding is widely thought to be critical for recognition between the prospective symbiotic partners. This hypothesis has been strongly supported by genetic evidence even though such binding specificity has not been demonstrated. The best examples are from the pea-*R. leguminosarum* symbiosis where bacterial *nod* gene mutants that lead to changed Nod factor composition or structure exhibited genotype-specific nodulation (Firmin et al., 1993; Bloemberg et al., 1995). This alteration of host range corresponds to allelic variations at the *Sym2/Sym37/PsK1* locus, an orthologous region of *NFR1* that contains a cluster of LysM receptor kinases (Zhukov et al., 2008; Li et al., 2011). In this case, allelic variation coupled with gene duplication and diversification contribute to alterations in symbiotic compatibility.

Nod factor recognition presumably plays a more critical role in determining host range at species level, which has been best illustrated on the bacterial side. However, natural polymorphisms in Nod-factor receptors that are responsible for nodulation specificity between different legumes have not been well studied at the genetic level, simply because the plants cannot be interbred. Nevertheless, transferring *NFR1* and *NFR5* of *L. japonicus* into *M. truncatula* enables nodulation of the transformants by the *L. japonicus* symbiont *Mesorhizobium loti* (Radutoiu et al., 2007).

## Specificity Mediated by Perception of Rhizobial Exopolysaccharides

In addition to Nod factors, rhizobial surface polysaccharides such as exopolysaccharides (EPS), lipopolysaccharides (LPS), and capsular polysaccharides (KPS) are also thought to be important for establishing symbiotic relationships (Fraysse et al., 2003; Becker et al., 2005; Jones et al., 2007; Gibson et al., 2008). These surface components are proposed to be able to suppress plant defense, but their active roles in promoting bacterial infection and nodulation remain elusive and are dependent on the specific interactions studied.

Exopolysaccharides have been shown to be required for rhizobial infection in multiple symbiotic interactions. This has been best illustrated in the *Sinorhizobium-Medicago* symbiosis, in which succinoglycan, a major EPS produced by *S. meliloti*, is required for the initiation and elongation of infection threads, and increased succinoglycan production enhances nodulation capacity (Leigh et al., 1985; Reinhold et al., 1994; Cheng and Walker, 1998; Jones, 2012). However, the symbiotic role of EPS is very complicated in the *Mesorhizobium-Lotus* interaction (Kelly et al., 2013). For instance, a subset of EPS mutants of *M. loti* R7A displayed severe nodulation deficiencies on *L. japonicus* and *L. corniculatus*, whereas other mutants formed effective nodules (Kelly et al., 2013). In particular, R7A mutants deficient in production of an acidic octasaccharide EPS were able to normally nodulate *L. japonicus*, while *exoU* mutants producing a truncated pentasaccharide EPS failed to invade the host. It was proposed that full-length EPS serves as a signal to compatible hosts to modulate plant defense responses and allow bacterial infection, and R7A mutants that make no EPS could avoid or suppress the plant surveillance system and therefore retain the ability to form nodules. In contrast, strains that produce modified or truncated EPS trigger plant defense responses resulting in block of infection (Kelly et al., 2013).

EPS production is common in rhizobial bacteria, and the composition of EPS produced by different species varies widely (Skorupska et al., 2006). Several studies have suggested the involvement of the EPS structures in determining infective specificity (Hotter and Scott, 1991; Kannenberg et al., 1992; Parniske et al., 1994; Kelly et al., 2013). Recently, an EPS receptor (EPR3) has been identified in *L. japonicus*, which is a cell surface-localized protein containing three extracellular LysM domains and an intracellular kinase domain (Kawaharada et al., 2015). EPR3 binds rhizobial EPS in a structurally specific manner. Interestingly, *Epr3* gene expression is contingent on Nod-factor signaling, suggesting that the bacterial entry to the host is controlled by two successive steps of receptor-mediated recognition of Nod factor and EPS signals (Kawaharada et al., 2015, 2017). The receptor-ligand interaction supports the notion that EPS recognition plays a role in regulation of symbiosis specificity. However, natural variation in host-range specificity that results from specific recognition between host receptors and strain-specific EPS has not been demonstrated in any legume-rhizobial interactions. It is noteworthy that acidic EPS of bacterial pathogens also promote infection to cause plant disease (Newman et al., 1994; Yu et al., 1999; Aslam et al., 2008; Beattie, 2011). Thus, rhizobial EPS might also be recognized by host immune receptors to induce defense responses that negatively regulate symbiosis development.

## Specificity Mediated by Host Innate Immunity

Symbiotic and pathogenic bacteria often produce similar signaling molecules to facilitate their invasion of the host (Deakin and Broughton, 2009). These molecules include conserved microbe-associated molecular patterns (MAMPs) and secreted effectors (D’Haeze and Holsters, 2004; Fauvart and Michiels, 2008; Deakin and Broughton, 2009; Soto et al., 2009; Downie, 2010; Wang et al., 2012; Okazaki et al., 2013). The host has evolved recognition mechanisms to distinguish between, and respond differently to, pathogens and symbionts (Bozsoki et al., 2017; Zipfel and Oldroyd, 2017). However, this discrimination is not always successful; as a result, recognition specificity frequently occurs in both pathogenic and symbiotic interactions. In the legume-rhizobial interaction, effector- or MAMP-triggered plant immunity mediated by host receptors also plays an important role in regulating host range of rhizobia (Yang et al., 2010; Wang et al., 2012; Faruque et al., 2015; Kawaharada et al., 2015; Tang et al., 2016).

Several dominant genes have been cloned in soybeans (e.g., *Rj2*, *Rfg1*, and *Rj4*) that restrict nodulation by specific rhizobial strains (Yang et al., 2010; Tang et al., 2016; Fan et al., 2017). In these cases, restriction of nodulation is controlled in a similar manner as ‘gene-for-gene’ resistance against plant pathogens. *Rj2* and *Rfg1* are allelic genes that encode a typical TIR-NBS-LRR resistance protein conferring resistance to multiple *B. japonicum* and *Sinorhizobium fredii* strains (Yang et al., 2010; Fan et al., 2017). *Rj4* encodes a thaumatin-like defense-related protein that restricts nodulation by specific strains of *B. elkanii* (Tang et al., 2016). The function of these genes is dependent on the bacterial type III secretion system and its secreted effectors (Krishnan et al., 2003; Okazaki et al., 2009; Yang et al., 2010; Tsukui et al., 2013; Tsurumaru et al., 2015; Tang et al., 2016; Yasuda et al., 2016). These studies indicate an important role of effector-triggered immunity in the regulation of nodulation specificity in soybeans. As discussed earlier, rhizobial Nod factors and surface polysaccharides could play a role in suppression of defense responses (Shaw and Long, 2003; D’Haeze and Holsters, 2004; Tellström et al., 2007; Jones et al., 2008; Liang et al., 2013; Cao et al., 2017), but these signaling events apparently are not strong enough to evade effector-trigged immunity in incompatible interactions.

Many rhizobial bacteria use the type III secretion system to deliver effectors into host cells to promote infection, and in certain situations, the delivered effector(s) are required for Nod-factor independent nodulation as demonstrated in the soybean-*B. elkanii* symbiosis (Deakin and Broughton, 2009; Okazaki et al., 2013, 2016). On the other hand, however, recognition of the effectors by host resistance genes triggers immune responses to restrict rhizobial infection. The nodulation resistance genes occur frequently in natural populations, raising a question why host evolve and maintain such seemingly unfavorable alleles. This could happen because of balancing selection, as the same alleles may also contribute to disease resistance against pathogens, considering that some rhizobial effectors are homologous to those secreted by bacterial pathogens (Dai et al., 2008; Kambara et al., 2009). Alternatively, legume may take advantage of *R* genes to exclude nodulation with less efficient nitrogen-fixing strains and selectively interact with strains with high nitrogen fixation efficiency, which is the case of the soybean *Rj4* allele.

A single dominant locus, called *NS1*, was also identified in *M. truncatula* that restricts nodulation by *S. meliloti* strain Rm41 (Liu et al., 2014). Unlike *R* gene-controlled host specificity in soybeans, which depends on bacterial type III secretion system, Rm41 strain lacks genes encoding such a system. It will be interesting to know what host gene(s) control this specificity and what bacterial signals are involved.

## Specificity in Nitrogen Fixation

Symbiotic specificity is not confined to the early recognition stages; incompatible host-strain combinations can lead to formation of nodules that are defective in nitrogen fixation (Fix-). For example, a screen of a core collection of *Medicago* accessions using multiple *S. meliloti* strains showed that ∼40% of the plant-strain combinations produced nodules but failed to fix nitrogen (Liu et al., 2014). The Fix- phenotype was not due to a lack of infection but caused by bacteroid degradation after differentiation (Yang et al., 2017; Wang et al., 2017, 2018).

Host genetic control of nitrogen fixation specificity is very complicated in the *Medicago-Sinorhizobium* symbiosis, involving multiple linked loci with complex epistatic and allelic interactions. By using the residual heterozygous lines identified from a recombination inbred line population, Zhu and colleagues were able to clone two of the underlying genes, namely *NFS1* and *NFS2*, that regulate strain-specific nitrogen fixation concerning the *S. meliloti* strains Rm41 and A145 (Wang et al., 2017, 2018; Yang et al., 2017). *NFS1* and *NFS2* both encode nodule-specific cysteine-rich (NCR) peptides (Mergaert et al., 2003). The NFS1 and NFS2 peptides function to provoke bacterial cell death and early nodule senescence in an allele-specific and rhizobial strain-specific manner, and their function is dependent on host genetic background. NCRs were previously shown to be positive regulators of symbiotic development, essential for terminal bacterial differentiation and for maintenance of bacterial survival in the nodule cells (Van de Velde et al., 2010; Wang et al., 2010; Horváth et al., 2015; Kim et al., 2015). The discovery of NFS1 and NFS2 revealed a negative role that NCRs play in regulation of symbiotic persistence, and showed that NCRs are host determinants of symbiotic specificity in *M. truncatula* and possibly also in closely related legumes that subject their symbiotic bacteria to terminal differentiation.

The genomes of *M. truncatula* and closely related galegoid legumes contain a large number of NCR-encoding genes that are expressed exclusively in the infected nodule cells (Montiel et al., 2017). These *NCR* genes, similar to bacterial type III effectors or MAMPs, can play both positive and negative roles in symbiotic development and both roles are associated with the antimicrobial property of the peptides. On one hand, the host uses this antimicrobial strategy for promoting terminal bacteroid differentiation to enhance nitrogen fixation efficiency (Oono and Denison, 2010; Oono et al., 2010; Van de Velde et al., 2010; Wang et al., 2010). On the other hand, some rhizobial strains cannot survive the antibacterial activity of certain peptide isoforms. The vulnerability of particular bacterial strains in response to a peptide is contingent on the genetic constitution of the bacteria as well as the genetic background of the host. It was proposed that this host-strain adaptation drives the coevolution of both symbiotic partners, leading to the rapid amplification and diversification of the *NCR* genes in galegoid legumes (Wang et al., 2017; Yang et al., 2017).

Host-range specificity in the ability to fix nitrogen has also been documented in legumes (e.g., soybeans) where the bacteria undergo reversible differentiation. In soybeans, this type of incompatibility was associated with the induction of phytoalexin accumulation and hypersensitive reaction in the nodule cells (Parniske et al., 1990). No *NCR* genes exist in the soybean genome, implying the involvement of novel genetic mechanisms that control this specificity. Work is in progress in our lab to identify the host genes that are involved.

## Conclusion and Future Perspectives

**FIGURE 1:**
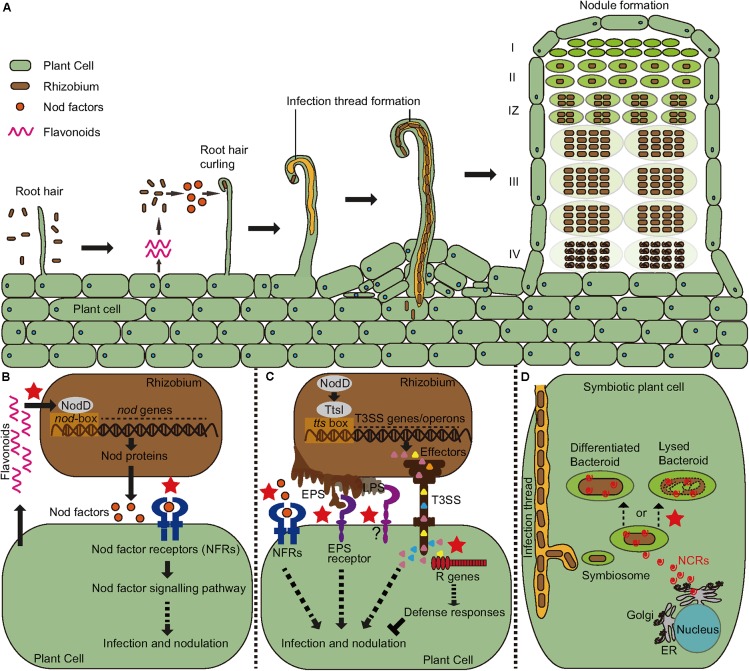
Symbiosis signaling and plant immunity involved in recognition specificity in the legume-rhizobial interactions (indicated by the red stars). **(A)** The process of infection and nodule development. A mature indeterminate nodule contains a meristem zone (I), an infection zone (II), an interzone (IZ), a nitrogen fixing zone (III), and a senescent zone (IV). **(B)** The host secretes flavonoids to induce the expression of bacterial nodulation (*nod*) gene through the activation of NodD proteins. The enzymes encoded by the *nod* genes lead to the synthesis of Nod factors (NF) that are recognized by host Nod factor receptors (NFRs). Recognition specificity occurs both between Flavonoids and NodDs and between NF and NFRs. **(C)** In addition to NF signaling, bacteria also produce extracellular polysaccharides (EPS) and type III effectors to facilitate their infection in compatible interactions, but these molecules may also induce immune responses causing resistance to infection in incompatible interactions. **(D)** Certain legumes such as *Medicago* encode antimicrobial nodule-specific cysteine-rich (NCR) peptides to drive their bacterial partners to terminal differentiation that is required for nitrogen fixation. However, some rhizobial strains cannot survive the antibacterial activity of certain peptide isoforms, leading to formation of nodules defective in nitrogen fixation.

Specificity in the legume-rhizobial symbiosis results from a suite of signal exchanges between the two symbiotic partners (summarized in **Figure 1**). Recent studies have just begun to reveal the underlying molecular mechanisms that regulate this specificity, and there are many challenging questions waiting to be answered. Effector-triggered immunity has been shown to be an important factor in determining host range of rhizobia in soybeans but the cognate effectors have not been clearly defined. In addition, what are the genes that control nodulation specificity in the *Medicago-Sinorhizobium* interaction where the bacterial partner lacks the type III secretion system? Cloning and characterization of the *NS1* locus in *M. truncatula* (Liu et al., 2014) will provide novel insights into this question. We now know that NCR peptides regulate nitrogen fixation specificity in *Medicago* and possibly in other closely related legumes, but we lack mechanistic understanding of how these peptides work. Do the pro- and anti-symbiotic peptides interact with the same bacterial targets? How do the amino-acid substitutions affect the peptide structure and function? How is nitrogen fixation specificity regulated in the NCR-lacking legumes such as soybeans where bacteria undergo reversible differentiation? These are just a handful of outstanding questions that need to be addressed. Answering these questions will certainly enrich our knowledge of how specificity is controlled and allow us to use such knowledge to develop tools for genetic improvement of symbiotic nitrogen fixation in legumes.

## Author Contributions

All authors listed have made a substantial, direct and intellectual contribution to the work, and approved it for publication.

## Conflict of Interest Statement

The authors declare that the research was conducted in the absence of any commercial or financial relationships that could be construed as a potential conflict of interest.
